# Acute Calcific Tendinitis of the Longus Colli: A Case Report and Review of the Literature

**DOI:** 10.7759/cureus.60409

**Published:** 2024-05-16

**Authors:** Moiud Mohyeldin, Harendra Singh, Mijjal Shrestha, Mauricio Leitao, Jai Kumar

**Affiliations:** 1 Internal Medicine, BronxCare Health System, Bronx, USA

**Keywords:** cervical spine, acute calcific tendonitis of the longus colli, calcium hydroxyapatite deposition, neck pain, retropharyngeal tendinitis, longus colli muscle, acute calcific tendinitis

## Abstract

Acute calcific tendinitis of the longus colli (ACTLC) is a rare, self-resolving condition caused by calcium hydroxyapatite crystal deposition in the longus colli muscle tendons. We present a case of a 46-year-old female with a history of hypertension who presented with right-sided neck pain, worsening abdominal pain, nausea, bloody emesis, and generalized body aches in the context of recent alcohol use. Physical examination revealed neck pain with limited range of motion, induration, and tenderness in the right and posterior neck areas. Laboratory findings showed elevated white cell count, inflammatory markers, and metabolic acidosis with an elevated anion gap and lactic acid level. Computed tomography (CT) of the neck with contrast demonstrated amorphous calcification in the longus colli tendons and retropharyngeal effusion, consistent with the diagnosis of ACTLC. The patient was treated with nonsteroidal anti-inflammatory drugs (NSAIDs) and supportive care, leading to symptom resolution. This case highlights the importance of considering ACTLC in the differential diagnosis of acute neck pain and the role of CT imaging in establishing the diagnosis. Prompt recognition and appropriate management of ACTLC can prevent unnecessary interventions and lead to improved patient outcomes.

## Introduction

Acute calcific tendinitis of the longus colli (ACTLC) is a rare, self-resolving inflammatory process caused by calcium hydroxyapatite crystal deposition in the tendons of the longus colli muscle [1-3]. The presence of these crystals triggers an inflammatory reaction resulting in tendinitis and reactive retropharyngeal fluid collection, which can be misinterpreted as an abscess on imaging [4]. Although the exact cause of ACTLC is unknown, it is thought to be preceded by repetitive trauma, degenerative cervical disorders, osteoarthritis, tissue necrosis, renal failure, or vascular disease.

The longus colli muscle, one of the four deep cervical flexor muscles, is composed of superior oblique, vertical, and inferior oblique fibers. It is located on the anterior surface of the vertebral column, spanning from the atlas to the third thoracic vertebra. This muscle is responsible for cervical flexion, ipsilateral flexion, and some rotational movement, with the superior oblique fibers being the most susceptible to calcific deposits [1,2].

ACTLC typically affects young adults between the ages of 30 and 60, with no ethnic or gender predilection. An epidemiologic study published in 2013 estimated an annual incidence of 0.5 cases per 100,000 person-years. Acute symptoms include neck pain, neck stiffness, dysphagia, odynophagia, mild fever, and decreased neck mobility, along with potential elevations in erythrocyte sedimentation rate (ESR), C-reactive protein (CRP), and white blood cell count [1,2].

Computed tomography (CT) is the gold standard for diagnosis, identifying prevertebral edema and longus colli calcium hydroxyapatite crystal deposits. In contrast, magnetic resonance imaging (MRI) can identify fluid effusion and prevertebral edema but not calcium deposits, making CT the most sensitive modality for diagnosing prevertebral calcification and differentiating between retropharyngeal tendinitis and retropharyngeal abscess [3].

Treatment typically involves supportive care combined with nonsteroidal anti-inflammatory drugs (NSAIDs), which can provide immediate relief within 48-72 hours, although recovery may occasionally require up to two weeks. Calcium deposits often resolve within a few weeks. In cases of severe symptoms, a course of corticosteroid medication may be initiated [4].

## Case presentation

A 46-year-old female presented with a seven-day history of right-sided neck pain of moderate severity, accompanied by a one-day history of worsening abdominal pain, nausea, and bloody emesis, as well as generalized body aches, all occurring in the context of recent alcohol use. Her past medical history was notable for hypertension. Upon examination, the patient's vital signs were as follows: blood pressure 132/84 mmHg, heart rate 126 beats/min, respiratory rate 20 breaths/min, oxygen saturation 97% on room air, and temperature 36.6 degrees Celsius. The patient exhibited neck pain with limitation of motion, with induration and tenderness observed in the right neck and posterior neck areas. Notably, there was no tenderness in the temporo-mandibular joint, intact dentition, normal palate and tonsils, no masses or lesions on the posterior pharynx wall or tongue, and no evidence of lymphadenopathy or thyroid enlargement. A facial examination revealed no abnormalities. Cardiorespiratory, abdominal, neurological, and musculoskeletal examinations were all within normal limits.

Laboratory findings indicated an elevated white cell count of 13.9k/μL, with a neutrophil predominance (neutrophil: 90.4%). Elevated inflammatory markers included an ESR of 26 mm/hr and CRP of 15.44 mg/L. Blood gas analysis revealed metabolic acidosis (pH: 7.189; pCO2: 24.7 mmHg; Bicarbonate: 8 mEq/L) with an elevated anion gap of 26 and a lactic acid level of 5.1. Additionally, serum blood urea nitrogen and creatinine were elevated at 36 mg/dL and 2 mg/dL, respectively.

**Figure 1:**
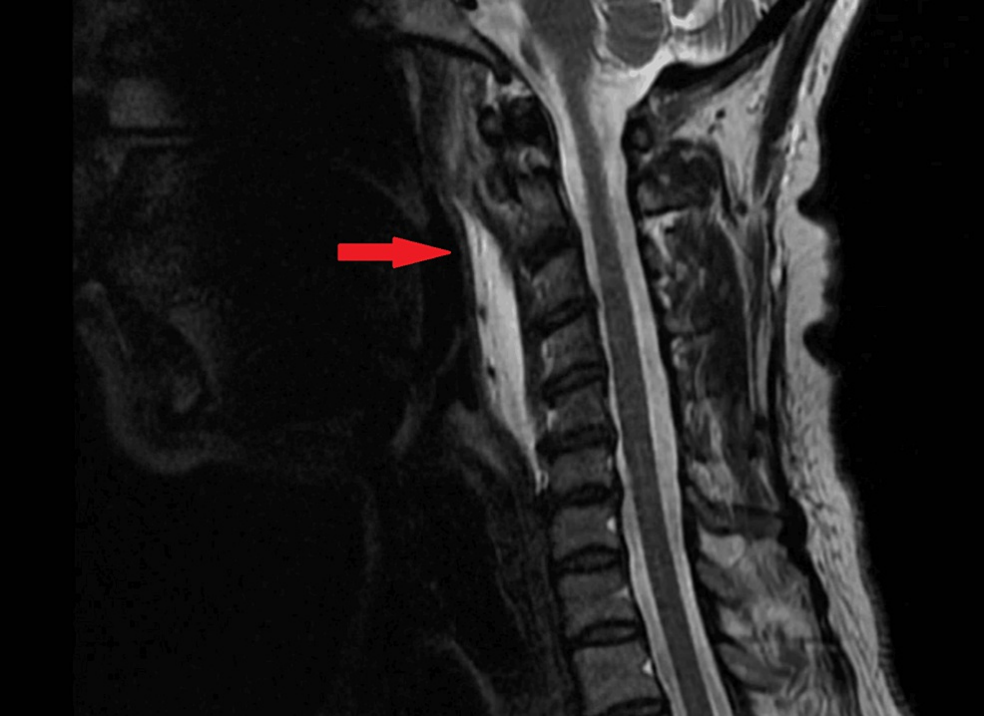
Magnetic resonance imaging (MRI), coronal T2-weighted image of the neck Retropharyngeal effusion (red arrow)

**Figure 2:**
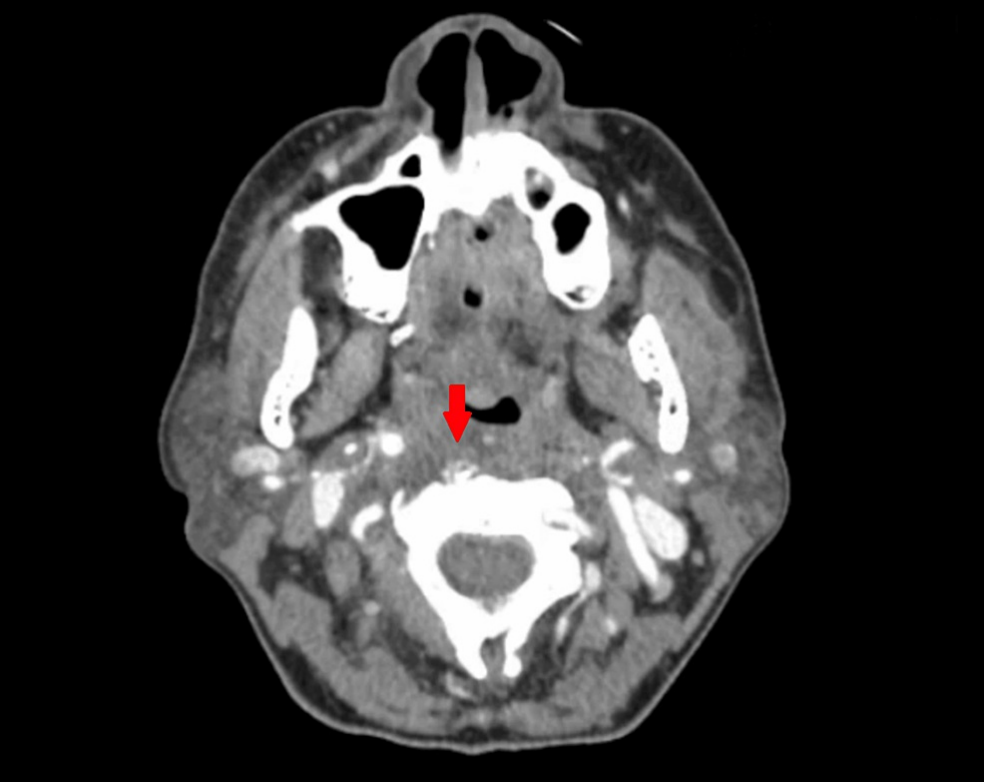
Contrast-enhanced computed tomography (CT) of the neck (axial view) Amorphous calcification of the superior fibers of the longus colli tendons (red arrow)

**Figure 3:**
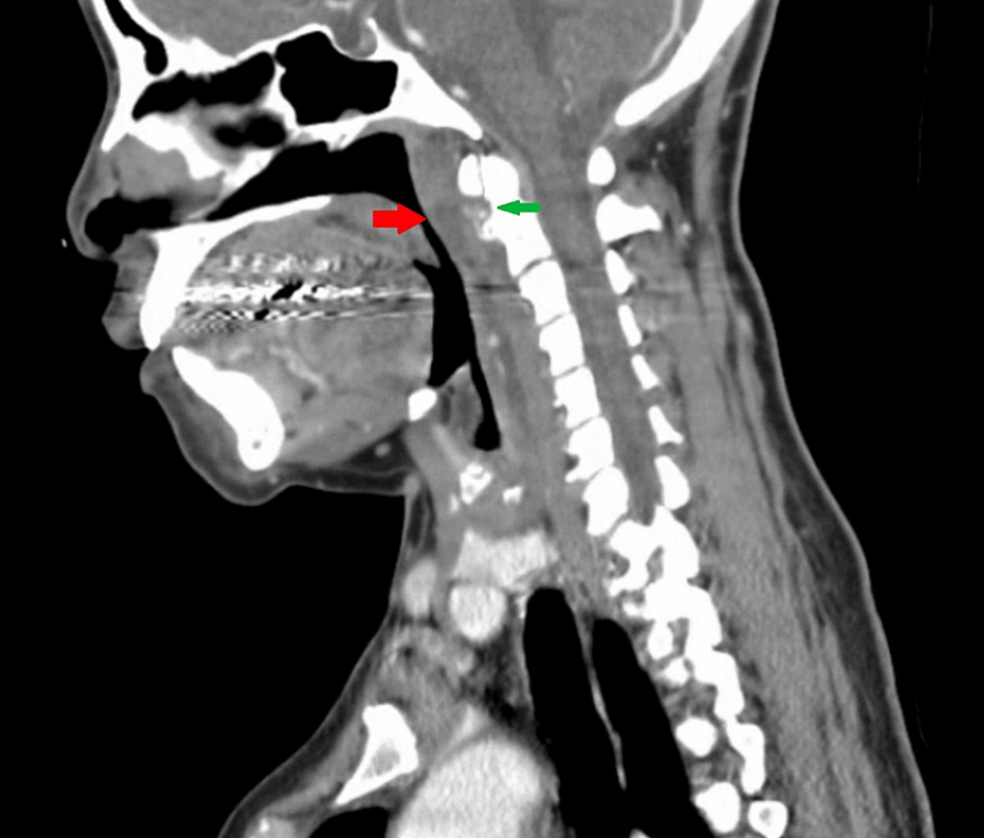
Contrast-enhanced computed tomography (CT) of the neck (sagittal view) Retropharyngeal effusion extending from C1 to C6 (red arrow); Amorphous calcification of the superior fibers of the longus colli tendons (green arrow)

In the emergency department, the patient was diagnosed with starvation ketoacidosis, and renal function improved with serum creatinine down-trending to 0.5 mg/dL. An MRI was conducted to investigate persistent neck pain, revealing retropharyngeal effusion and small effusions in atlantoaxial joints bilaterally (Figure 1). A subsequent contrast-enhanced CT of the neck revealed amorphous calcification approximating the superior fibers of the longus colli tendons, accompanied by retropharyngeal effusion extending from C1 to C6 caudally (Figure 2, Figure 3).

The patient was initially treated with cyclobenzaprine and ketorolac for pain management. Consultation with Ear, Nose, and Throat (ENT) services resulted in the recommendation of empiric antibiotics for possible retropharyngeal cellulitis. The patient was started on intravenous ceftriaxone.

During the hospital course, the patient's starvation ketoacidosis resolved with fluid resuscitation. Alcohol withdrawal was managed with supportive care. Acute kidney injury and hypophosphatemia improved with appropriate management. The patient received physical therapy for neck pain and limited range of motion.

At the time of discharge, the patient's neck pain had improved significantly. The patient was discharged on oral antibiotics and follow-up with ENT as an outpatient. The patient was also advised to follow up with primary care for the management of hypertension and alcohol use disorder. Social work was involved in discharge planning to a skilled nursing facility for further rehabilitation.

## Discussion

First described by Hartley in 1964, acute calcific tendinitis of longus colli (ACTLC) is an acute or sub-acute reactive, self-limiting, inflammatory response to deposition of amorphous calcium hydroxyapatite crystals in the tendons of the longus colli muscle [1-3]. The presence of these crystals incites an inflammatory response resulting in tendinitis and a reactive retropharyngeal fluid collection that can be misinterpreted as an abscess on imaging [4]. Acute inflammatory reaction is caused by the rupture and release of hydroxyapatite crystals into the surrounding soft tissue, whereas localized calcium deposition serves as a temporary attempt to make up for diminished tendon strength [5,6].

Although the exact cause of ACTLC is unknown, it is thought that calcium and hydroxyapatite crystal deposition precedes recurrent stress, ischemia, necrosis, and longus colli tendon degeneration. Known risk factors for developing ACTLC include repetitive trauma, degenerative cervical disorders, osteoarthritis, tissue necrosis, renal failure, or vascular disease [7].

Superior oblique, vertical, and inferior oblique fibers form the longus colli muscle, one of the four deep cervical flexor muscles. It is applied to the anterior surface of the vertebral column, between the atlas and the third thoracic vertebra [8]. This muscle is responsible for cervical flexion, ipsilateral flexion, and some rotational movement. The superior oblique fibers are the most susceptible to calcific deposits [9] and likely are the cause of common symptoms of pain during the lateral flexion of the neck.

It typically affects young adults between the ages of 30 and 60, with a documented age distribution ranging from 21 to 80. There is no ethnic or gender predilection. An epidemiologic study that was published in 2013 concluded that ACTLC is not as uncommon as the literature suggests, estimating an annual incidence of 0.5 cases per 100,000 person-years [10].

Acute symptoms include neck pain, neck stiffness, dysphagia, odynophagia, mild fever, and decreased neck mobility. There may be an increased ESR, CRP, and white blood cell count [5].

Computed tomography (CT) is considered the gold standard for diagnosing acute calcific tendinitis of the longus colli (ACTLC) because it can identify both prevertebral edema and calcium hydroxyapatite crystal deposition in the longus colli tendons. In contrast, magnetic resonance imaging (MRI) can identify fluid effusion and prevertebral edema but not calcium deposits. As a result, when diagnosing prevertebral calcification, CT is more sensitive than MRI. Furthermore, the most sensitive radiological technique for differentiating between retropharyngeal tendinitis and retropharyngeal abscess is computed tomography (CT) [11,12].

Supportive care combined with NSAID medication can result in a successful course of treatment. While most patients have immediate relief from NSAIDs within 48-72 hours, recovery may occasionally take up to two weeks. Calcium deposits often go away within a few weeks. A course of corticosteroid medication may be initiated if symptoms are severe [13-15].

## Conclusions

Acute calcific tendinitis of the longus colli (ACTLC) is an uncommon, self-limiting condition that should be considered in the differential diagnosis of acute neck pain, particularly when accompanied by dysphagia, odynophagia, and retropharyngeal edema. This case report highlights the importance of a thorough clinical evaluation and appropriate imaging studies in diagnosing ACTLC. The patient's presentation of neck pain, limited range of motion, and elevated inflammatory markers, along with the characteristic CT findings of amorphous calcification in the longus colli tendons and retropharyngeal effusion, led to the diagnosis of ACTLC. Although ACTLC is a self-limiting condition, prompt diagnosis and appropriate management are crucial to alleviate symptoms and prevent unnecessary interventions. Conservative treatment with NSAIDs and supportive care is usually sufficient, with most patients experiencing significant relief within 48-72 hours. In rare cases of severe symptoms, a short course of corticosteroids may be considered.

This case underscores the importance of considering ACTLC in the differential diagnosis of acute neck pain and the role of CT imaging in establishing the diagnosis. Increased awareness of this condition among healthcare professionals can lead to earlier recognition, appropriate management, and improved patient outcomes. Further research is needed to better understand the epidemiology, risk factors, and long-term outcomes of ACTLC.
